# The Relationship between Serum Ferritin Levels and Insulin Resistance in Pre- and Postmenopausal Korean Women: KNHANES 2007–2010

**DOI:** 10.1371/journal.pone.0157934

**Published:** 2016-06-23

**Authors:** Min Kyoung Kim, Seung Joo Chon, Yeon Soo Jung, Bo Ok Kim, Eun Bee Noe, Bo Hyon Yun, SiHyun Cho, Young Sik Choi, Byung Seok Lee, Seok Kyo Seo

**Affiliations:** 1 Department of Obstetrics and Gynecology, Severance Hospital, Yonsei University College of Medicine, Seoul, Republic of Korea; 2 Department of Obstetrics and Gynecology, Gil Hospital, Gachon University College of Medicine, Incheon, Republic of Korea; 3 Department of Obstetrics and Gynecology, Wonju Severance Christian Hospital, Yonsei University Wonju College of Medicine, Wonju, Republic of Korea; 4 Biostatistics Collaboration Unit, Yonsei University College of Medicine, Seoul, Republic of Korea; 5 Seoul Rachel Fertility Center, Seoul, Republic of Korea; 6 Department of Obstetrics and Gynecology, Gangnam Severance Hospital, Yonsei University College of Medicine, Seoul, Republic of Korea; 7 Institute of Women’s Life Medical Science, Yonsei University College of Medicine, Seoul, Republic of Korea; College of Tropical Agriculture and Human Resources, University of Hawaii, UNITED STATES

## Abstract

**Background:**

Serum ferritin levels increase in postmenopausal women, and they are reported to be linked to major health problems. Here, we investigated the association between serum ferritin levels and insulin resistance (IR) in postmenopausal women.

**Methods:**

A total of 6632 healthy Korean women (4357 premenopausal and 2275 postmenopausal) who participated in the Korean National Health and Nutrition Examination Survey (KNHANES) in 2007–2010 were enrolled in the study. Serum ferritin values were divided into six groups for the premenopausal and postmenopausal groups. IR and obesity indices were evaluated according to the six serum ferritin groups. Statistical analysis was carried out using SAS software, version 9.2 (SAS Institute Inc., Cary, NC, USA).

**Results:**

The association between the IR indices and ferritin groups had a higher level of statistical significance in the postmenopausal group than in the premenopausal group. In addition, for the postmenopausal group, the estimates increased significantly in the sixth ferritin group compared to those in the first ferritin group. However, the association between the obesity indices and ferritin levels was not significantly different between the premenopausal and postmenopausal groups.

**Conclusion:**

Elevated serum ferritin levels were associated with an increased risk of insulin resistance in postmenopausal women.

## Introduction

Iron is an important trace element that is required for transporting oxygen, oxidative phosphorylation, DNA biosynthesis, xenobiotic metabolism, and biological processes that involve the transfer of electrons in the human body [1,2]. Electron transfer occurs via oxidation-reduction reactions that cause iron to fluctuate between its ferric (+3) and ferrous (+2) states [3]. This fluctuation can cause toxicity if there is an excess of “labile iron”.

Serum ferritin is the most reliable marker of iron stores in the body. As women age and go through menopause, their bodies undergo several physiological changes, including those of serum ferritin levels. Serum ferritin levels increase in postmenopausal women as a result of the cessation of menstrual periods. Researchers have hypothesized that increased serum ferritin levels, including those that are within the normal physiological range, are linked to major health problems in postmenopausal women [4]. For example, iron is proposed to have a role in the pathogenesis of many diseases, such as IR, diabetes mellitus (DM), infection, cardiovascular diseases (CVD), and cancer [5].

IR is a physiological condition that is closely linked to metabolic syndrome and DM. The incidence of IR is increasing and early diagnosis has become important to better manage and prevent patients from developing more severe diseases. Generally, IR is diagnosed by measuring fasting insulin levels or glucose tolerance tests. However, patients do not regularly take those specific blood tests unless they have other underlying diseases or are diligent about having regular health check-ups. Most patients visit hospitals after suffering from symptoms of IR or developing other diseases. It is preferable to have a diagnostic marker that allows physicians to predict and prevent IR as early as possible.

Previous studies describe a positive relationship between serum ferritin levels, which is a marker for elevated iron stores, and IR in women and men regardless of age [6–10]. This information combined with the fact that iron stores increase in postmenopausal women led to the following question: do postmenopausal women with increased serum ferritin levels have a greater risk of developing IR than premenopausal women? Therefore, we tested the hypothesis that there is an association between serum ferritin levels and IR in postmenopausal women due to elevated iron stores in pre- and postmenopausal women.

## Subjects and Methods

### Study population

The data used in this study are from the KNHANES, specifically the KNHANES IV (2007–2009) and the KNHANES V (2010), which were conducted by the Korean Ministry of Health and Welfare. KNHANES were each conducted using a rolling sample survey that involved a complex, stratified, multistage, probability-cluster survey of a representative sample of the non-institutionalized civilian population in South Korea. Individuals surveyed were randomly selected from 2300 households in 2007, 4600 households each in 2008 and 2009, and 3840 households in 2010. The sampling units were newly selected each year and did not overlap with previous samples. We included data from 2007–2010 in this study because insulin levels were not recorded from 2011. The survey consisted of three parts: a health interview survey, a health examination survey, and a nutrition survey. All surveys were conducted by trained interviewers who were not provided previous information about the survey participants. Standard direct physical examinations were conducted in mobile examination centers. Before the survey, the participants completed written informed consent and we received the anonymized data. The current study was conducted according to the tenants of the Declaration of Helsinki and was approved by the Institutional Review Board at Yonsei University Health System, Severance Hospital (4-2015-0208).

**Fig 1:**
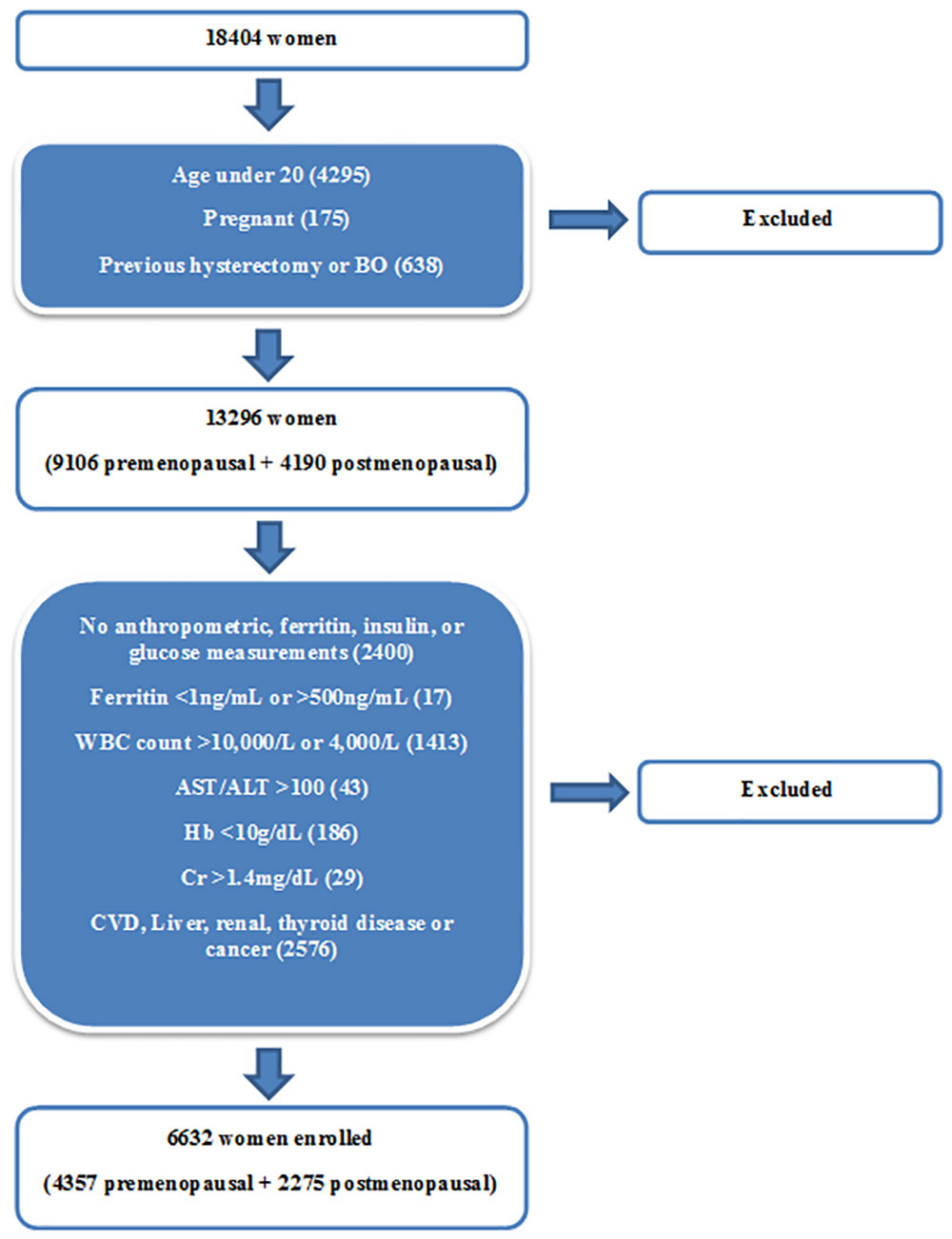
Flow chart of participant enrollment.

The exclusion criteria were similar to those of our previous study [11,12] (Fig 1). From the total of 18404 women, we initially excluded 5108 individuals who were younger than 20 years old, pregnant, or who had undergone a hysterectomy or bilateral oophorectomy (BO). Afterwards, 2400 women who did not have anthropometric measurements of height (Ht), waist circumference (WC), and weight, or provide blood samples for ferritin, fasting glucose, or insulin measurements were eliminated. Other exclusion criteria were aspartate aminotransferase (AST) or alanine aminotransferase (ALT) levels >100 IU/L, hemoglobin (Hb) levels <10 g/dL, creatinine (Cr) levels >1.4 mg/dL, white blood cell (WBC) counts >10,000/μl or <4,000/μl, and those who suffered from CVD, liver, renal, thyroid diseases or cancer. This eliminated disease category included inflammatory diseases (i.e. hepatitis, liver cirrhosis, etc.) to eradicate the possibility of having analytical error from serum ferritin levels being affected by inflammation. We also did not include 17 individuals with serum ferritin levels <1 ng/mL and >500 ng/mL because they could have ferritin-related diseases, such as hemochromatosis or sickle cell disease. Ultimately, there were 6632 participants (4357 premenopausal women and 2275 postmenopausal women) in the present study.

### Variable measurements

The confounding variables included age, smoking, alcohol consumption, exercise, WBC counts, Hb levels, Cr levels, total cholesterol levels, hormone replacement therapy (HRT), and DM treatment. Subjects were classified as never smokers, past smokers, occasional smokers, and current smokers (i.e., they smoked at least one cigarette per day during the previous 12 months). Alcohol consumption was categorized into six groups depending on drinking history during the previous 12 months. Exercise levels were divided into eight groups that depended on the number of days the subjects walked during the week. The subjects were also classified according to whether they had any kind of HRT or DM treatment.

Anthropometric measurements, such as Ht, WC, and weight, were taken while the subjects were wearing light clothing without shoes. Blood samples were taken in the early morning after fasting overnight. Plasma concentrations of Hb, ferritin, insulin, fasting glucose, Cr, total cholesterol, AST, and ALT levels were measured according to routine biochemical laboratory protocols. Ferritin was measured via chemiluminescent immunoassay using an ADVIA Centaur system (Siemens, Malvern, PA, USA) through February 15, 2008 and an immunoradiometric assay was performed with a 1470 WIZARD gamma counter (Perkin Elmer, Turku, Finland) from February 16, 2008 until 2010. The clinical analyses were all performed at a laboratory certified by the Korean Ministry of Health and Welfare (Neodin Medical Institute).

### Criteria and definitions

Menopause is defined as 12 months of amenorrhea following the final menstrual period [13]. The postmenopausal status in this study was defined as the subjects’ self-reported cessation of menstruation for more than 1 year. We excluded those with surgical menopause (i.e., individuals who had a hysterectomy or BO). Serum ferritin levels higher than 500 ng/mL were used as a marker for iron overload diseases [14,15]. Values of serum ferritin were divided into six groups for the premenopausal and postmenopausal women. For IR indices, we used insulin, fasting glucose levels, homeostasis model assessment IR (HOMA-IR), and obesity indices, including BMI ≥25 kg/m^2^, WC ≥85 cm, and waist-to-height ratio (WC/Ht ≥0.51). HOMA-IR was calculated as follows: HOMA-IR = fasting glucose × insulin/405 mg/dL.

### Statistical analysis

Data are expressed as means ± standard deviation for continuous variables and in percentages (%) for categorical variables. All IR indices, including obesity indices, were compared according to ferritin groups using one-way analysis of variance (ANOVA) and a Chi-square test. A trend test was applied to compare the continuous variables (i.e., insulin, fasting glucose levels, and HOMA-IR) according to ferritin groups. In order to compare IR indices according to ferritin groups after adjustment for confounding variables, multiple linear logistic regression and multiple linear regression were used to analyze the subjects. The covariates for the adjusted calculation were as described above in the ‘variable measurements’ subsection. Data analysis was carried out using SAS software, version 9.2 (SAS Institute Inc., Cary, NC, USA). We considered a p-value of <0.05 statistically significant.

## Results

Baseline clinical and laboratory characteristics of the total study population according to ferritin groups are described in Tables 1 and 2. The abnormal values of BMI, WC, and WC/Ht were significantly associated with increasing ferritin levels in postmenopausal women. Although all IR indices did not show significantly increasing trend according to the ferritin levels, each ferritin group had significantly different values of IR indices in postmenopausal women.

**Table 1:** Baseline characteristics of premenopausal women according to serum ferritin groups.

		1 (n = 727)F (n = 7	2 (n = 726)9.57<F≤17.32	3 (n = 727)17.32<F≤25.86	4 (n = 725)25.86<F≤36.62	5 (n = 727)36.62<F≤52.93	6 (n = 725)F>52.93	*p*-value†	*p*-value‡
**Ferritin (ng/mL)**		6.2±2.05	13.42±2.26	21.37±2.47	31.14±3.18	44.02±4.6	78.02±29.19		
**Age**		**37.19±8.19**	**35.75±8.23**	**35.64±8.4**	**35.72±8.27**	**36.09±8.16**	**35.91±8.72**	0.0033*	0.0011*
**Ht (cm)**		**158.61±5.83**	**159.24±5.78**	**159.63±5.34**	**159.43±5.37**	**159.1±5.35**	**159.17±5.34**	0.0130*	0.0018*
**BMI (kg/m**^**2**^**)**		**22.43±3.38**	**22.28±3.47**	**22.58±3.55**	**22.37±3.8**	**22.49±3.51**	**22.9±3.84**	0.0205*	0.8424
**WC (cm)**		**75.41±8.6**	**74.9±8.73**	**76±9.15**	**75.14±9.01**	**75.65±8.88**	**77.57±10.2**	<.0001*	0.8470
**WC/Ht**		**0.476±0.057**	**0.471±0.056**	**0.477±0.059**	**0.472±0.058**	**0.476±0.058**	**0.488±0.065**	<.0001*	0.4737
**Fasting glucose (mg/dl)**		**91.59±17.1**	**89.74±11.63**	**89.88±13.92**	**89.81±10.43**	**91.7±17.75**	**93.88±25.62**	<.0001*	0.0633
**Insulin (μIU/mL)**		9.94±4.04	9.57±4.14	9.7±5.18	9.37±4.18	9.77±8.11	9.74±3.98	0.3901	0.0611
**HOMA-IR (mg/dl)**		2.26±1.04	2.15±1.11	2.18±1.36	2.1±1.09	2.25±2.26	2.28±1.18	0.0897	0.0581
**WBC (Thous/μl)**		**5.72±1.14**	**5.89±1.25**	**5.81±1.26**	**5.92±1.25**	**5.99±1.25**	**6.11±1.31**	<.0001*	0.0128*
**Hb (g/dl)**		**12.03±0.996**	**12.99±0.796**	**13.1±0.814**	**13.18±0.768**	**13.24±0.802**	**13.32±0.866**	<.0001*	<.0001*
**Cr (mg/dl)**		0.741±0.105	0.743±0.115	0.734±0.109	0.734±0.111	0.728±0.104	0.73±0.109	0.0657	0.0996
**Total cholesterol (mg/dl)**		**181.18±31.35**	**177.27±30.18**	**174.63±28.68**	**176.55±30.76**	**176.46±31.71**	**179.76±34.3**	0.0007*	0.0014*
**AST (IU/L)**		**17.34±4.54**	**17.46±4.89**	**17.68±5.27**	**17.79±5.12**	**18.24±6.15**	**19.75±7.63**	<.0001*	0.0943
**ALT (IU/L)**		**13.94±6.43**	**14.3±7.11**	**15.03±8.84**	**15.18±7.82**	**16.44±9.7**	**19.03±12.84**	<.0001*	0.0030*
**BMI**	**<25**	576(79.23)	586(80.72)	564(77.58)	571(78.76)	576(79.23)	546(75.31)	0.1974	0.0733
	**≥25**	151(20.77)	140(19.28)	163(22.42)	154(21.24)	151(20.77)	179(24.69)		
**WC**	**<85**	**624(85.83)**	**633(87.19)**	**617(84.87)**	**625(86.21)**	**624(85.83)**	**572(78.9)**	0.0001*	0.0008*
	**≥85**	**103(14.17)**	**93(12.81)**	**110(15.13)**	**100(13.79)**	**103(14.17)**	**153(21.1)**		
**WC/Ht**	**<0.51**	**535(73.59)**	**563(77.55)**	**543(74.69)**	**549(75.72)**	**537(73.87)**	**489(67.45)**	0.0005*	0.0029*
	**≥0.51**	**192(26.41)**	**163(22.45)**	**184(25.31)**	**176(24.28)**	**190(26.13)**	**236(32.55)**		

# F = Ferritin, WC = Waist circumference, Ht = height

*p*-value†: Chi-square test, ANOVA

*p*-value‡: Trend test

* indicates statistically significant values (*p*<0.05)

**Table 2:** Baseline characteristics of postmenopausal women according to serum ferritin groups.

		1 (n = 380)	2 (n = 379)	3 (n = 379)	4 (n = 379)	5 (n = 379)	6 (n = 379)	*p*-value†	*p*-value‡
		F≤29.92	29.92<F≤43.9	43.9<F≤56	56<F≤72.73	72.73<F≤98.21	F>98.21		
**Ferritin (ng/mL)**		19.7±7.03	37.61±3.91	49.86±3.5	64.4±4.92	84.25±7.12	144.24±55.04		
**Age**		**62.35±9.51**	**62.32±8.94**	**61.64±9.2**	**62.61±9.44**	**63.6±9.44**	**65.13±9.65**	<.0001*	0.9667
**Ht (cm)**		**152.1±6.46**	**153.13±6.38**	**153.27±5.92**	**153.18±6.3**	**152.49±5.84**	**152.49±6.15**	0.0432*	0.0171*
**BMI (kg/m**^**2**^**)**		**23±3.42**	**23.31±3.43**	**23.2±2.98**	**23.38±3.41**	**23.51±3.4**	**23.93±3.64**	0.0053*	0.1847
**WC (cm)**		**78.92±9.01**	**80.56±8.93**	**80.14±8.5**	**81.37±8.69**	**81.75±8.95**	**83.62±9.21**	<.0001*	0.0007*
**WC/Ht**		**0.52±0.062**	**0.527±0.059**	**0.523±0.057**	**0.532±0.058**	**0.537±0.06**	**0.549±0.065**	<0001*	0.0167*
**Fasting glucose (mg/dl)**		**95.39±17.13**	**95.99±19.4**	**95.8±17.62**	**98.35±24.92**	**100.1±22.89**	**103.8±29.21**	<.0001*	0.0902
**Insulin (μIU/mL)**		**9.22±9.4**	**9.36±4.59**	**9.05±3.89**	**9.44±4.02**	**9.84±5.57**	**10.99±6.72**	<.0001*	0.8064
**HOMA-IR (mg/dl)**		**2.25±3.25**	**2.25±1.32**	**2.19±1.3**	**2.32±1.27**	**2.49±1.83**	**2.93±2.75**	<.0001*	0.7501
**WBC (Thous/μl)**		**5.69±1.2**	**5.68±1.21**	**5.73±1.23**	**5.74±1.16**	**5.9±1.29**	**6.02±1.39**	0.0004*	0.4570
**Hb (g/dl)**		**12.79±0.979**	**13±0.82**	**13.18±0.838**	**13.13±0.912**	**13.26±0.936**	**13.36±0.917**	<.0001*	<0001*
**Cr (mg/dl)**		0.75±0.132	0.741±0.121	0.733±0.121	0.738±0.123	0.739±0.127	0.749±0.133	0.3934	0.1405
**Total cholesterol (mg/dl)**		201.76±34.43	202.61±33.57	204.06±34.86	205.73±37.02	205.18±33.44	207.29±36.3	0.2617	0.0967
**AST (IU/L)**		**21.92±6.52**	**21.68±6.12**	**21.76±6.49**	**22.31±6.41**	**22.89±6.76**	**24.81±9.81**	<.0001*	0.4437
**ALT (IU/L)**		**17.44±7.91**	**17.74±7.81**	**18.29±8.57**	**19.85±9.55**	**20.16±10.36**	**22.51±12.17**	<.0001*	0.0004*
**BMI**	**<25**	**280(73.68)**	**261(68.87)**	**279(73.61)**	**253(66.75)**	**258(68.07)**	**238(62.8)**	0.0083*	0.0014*
	**≥25**	**100(26.32)**	**118(31.13)**	**100(26.39)**	**126(33.25)**	**121(31.93)**	**141(37.2)**		
**WC**	**<85**	**285(75)**	**258(68.07)**	**274(72.3)**	**252(66.49)**	**248(65.44)**	**221(58.31)**	<.0001*	<.0001*
	**≥85**	**95(25)**	**121(31.93)**	**105(27.7)**	**127(33.51)**	**131(34.56)**	**158(41.69)**		
**WC/Ht**	**<0.51**	**166(43.68)**	**157(41.42)**	**162(42.74)**	**142(37.47)**	**122(32.19)**	**105(27.7)**	<.0001*	<.0001*
	**≥0.51**	**214(56.32)**	**222(58.58)**	**217(57.26)**	**237(62.53)**	**257(67.81)**	**274(72.3)**		

# F = Ferritin, WC = Waist circumference, Ht = height

*p*-value†: Chi-square test, ANOVA

*p*-value‡: Trend test

* indicates statistically significant values (*p*<0.05)

**Fig 2:**
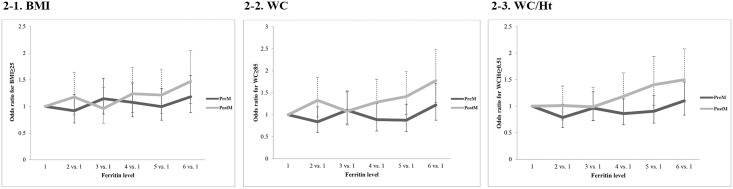
Odds ratio estimates for obesity indices according to serum ferritin groups after adjusting with confounders.

The subjects were divided into two groups, 4357 premenopausal and 2275 postmenopausal women, which were again categorized into six groups according to serum ferritin levels. Model 1 was adjusted for age only, and model 2 was adjusted for all the covariates. Both models were analyzed with multiple linear logistic regression. Tables 3, 4 and 5 and Fig 2 show the comparison of adjusted odds ratios (ORs) of BMI, WC, and WC/Ht by serum ferritin levels between the two groups. The association between the obesity indices and ferritin levels was not significantly different between the premenopausal and postmenopausal groups. However, postmenopausal women in both model 1 and 2 had significantly higher ORs in ferritin level 6 than in ferritin level 1 (BMI; p = 0.0011 (model 1), p = 0.0229 (model 2), WC; p<0.0001 (model 1), p = 0.0007 (model 2), and WC/Ht; p<0.0001 (model 1), p = 0.0163 (model 2). For premenopausal women, all model 1 statistics showed higher ORs in ferritin level 6 than in ferritin level 1 (BMI; p = 0.0407, WC; p = 0.0001, and WC/Ht; p = 0.0008).

**Table 3:** Adjusted odds ratios (95% confidence interval) for BMI (≥25) by serum ferritin groups.

	Premenopause	Postmenopause	*p*-value
	OR(95% CI); *p*-value	OR(95% CI); *p*-value	
Model 1			0.2331
Ferritin level 1	1	1	
Ferritin level 2	0.947(0.731–1.226); 0.6773	1.266(0.923–1.735); 0.1429	
Ferritin level 3	1.150(0.894–1.478); 0.2770	1.001(0.725–1.383); 0.9950	
Ferritin level 4	1.070(0.830–1.379); 0.6005	1.396(1.021–1.908); 0.0366	
Ferritin level 5	1.030(0.799–1.329); 0.8182	1.319(0.963–1.807); 0.0844	
Ferritin level 6	1.295(1.011–1.659); 0.0407	1.676(1.229–2.285); 0.0011	
Model 2			0.3929
Ferritin level 1	1	1	
Ferritin level 2	0.920(0.690–1.225); 0.5673	1.177(0.844–1.642); 0.3368	
Ferritin level 3	1.146(0.860–1.528); 0.3513	0.961(0.683–1.351); 0.8174	
Ferritin level 4	1.077(0.806–1.440); 0.6146	1.238(0.885–1.733); 0.2124	
Ferritin level 5	0.996(0.742–1.337); 0.9799	1.214(0.869–1.698); 0.2560	
Ferritin level 6	1.182(0.884–1.580); 0.2592	1.470(1.055–2.050); 0.0229	

**Table 4:** Adjusted odds ratios (95% confidence interval) for WC (≥85 cm) by serum ferritin groups.

	Premenopause	Postmenopause	*p*-value
	OR(95% CI); *p*-value	OR(95% CI); *p*-value	
Model 1			0.1713
Ferritin level 1	1	1	
Ferritin level 2	0.950(0.700–1.287); 0.7385	1.408(1.025–1.934); 0.0345	
Ferritin level 3	1.160(0.864–1.556); 0.3229	1.158(0.838–1.601); 0.3743	
Ferritin level 4	1.036(0.768–1.398); 0.8148	1.509(1.101–2.069); 0.0106	
Ferritin level 5	1.053(0.782–1.418); 0.7338	1.566(1.144–2.146); 0.0052	
Ferritin level 6	1.728(1.309–2.281); 0.0001	2.09(1.532–2.85); <.0001	
Model 2			0.1713
Ferritin level 1	1	1	
Ferritin level 2	0.840(0.599–1.179); 0.3133	1.326(0.950–1.851); 0.0975	
Ferritin level 3	1.102(0.788–1.540); 0.5709	1.079(0.767–1.519); 0.6613	
Ferritin level 4	0.888(0.631–1.252); 0.4989	1.285(0.915–1.804); 0.1481	
Ferritin level 5	0.876(0.620–1.237); 0.4525	1.414(1.011–1.976); 0.0427	
Ferritin level 6	1.221(0.876–1.703); 0.2386	1.776(1.275–2.475); 0.0007	

**Table 5:** Adjusted odds ratios (95% confidence interval) for WC/Ht (≥0.51) by serum ferritin groups

	Premenopause	Postmenopause	*p*-value
	OR(95% CI); *p*-value	OR(95% CI); *p*-value	
Model 1			0.3212
Ferritin level 1	1	1	
Ferritin level 2	0.883(0.690–1.131); 0.3261	1.099(0.821–1.470); 0.5261	
Ferritin level 3	1.049(0.823–1.337); 0.7001	1.064(0.796–1.423); 0.6744	
Ferritin level 4	0.985(0.772–1.257); 0.9037	1.291(0.962–1.732); 0.0884	
Ferritin level 5	1.069(0.840–1.360); 0.5900	1.584(1.175–2.137); 0.0026	
Ferritin level 6	1.496(1.182–1.892); 0.0008	1.875(1.380–2.548); <.0001	
Model 2			0.3554
Ferritin level 1	1	1	
Ferritin level 2	0.788(0.598–1.039); 0.0917	1.012(0.742–1.380); 0.9400	
Ferritin level 3	0.964(0.730–1.273); 0.7973	0.988(0.724–1.349); 0.9408	
Ferritin level 4	0.860(0.649–1.138); 0.2906	1.186(0.864–1.629); 0.2919	
Ferritin level 5	0.905(0.683–1.199); 0.4879	1.404(1.018–1.935); 0.0383	
Ferritin level 6	1.102(0.833–1.456); 0.4971	1.498(1.077–2.083); 0.0163	

BMI = body mass index, CI = confidence interval, OR = odds ratio, WC = Waist circumference, Ht = height

Model 1 & 2: Multiple linear logistic regression

Model 1 was adjusted for age

Model 2 was adjusted for age + covariates (alcohol consumption, smoking status, exercise levels, WBC, Hb, Cr, total cholesterol, HRT, and DM treatment)

Odds ratio estimates for BMI, WC, and WC/Ht after adjusting with confounders in postmenopausal women increase as serum ferritin levels increase. However, the numbers did not show clear trends in premenopausal women. (PreM; Premenopause, PostM; Postmenopause)

**Fig 3:**
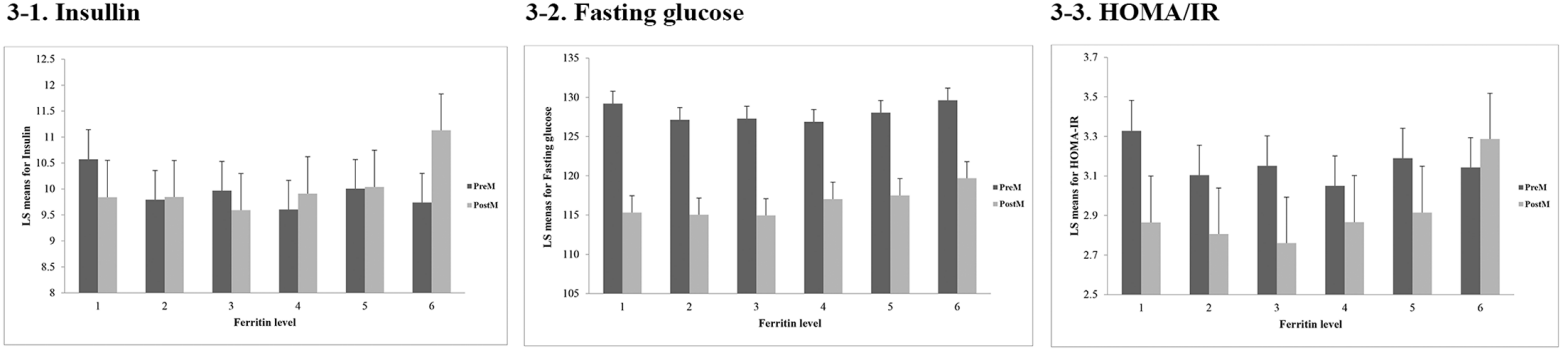
Least square means for insulin resistance indices according to serum ferritin groups after adjusting with confounders.

Tables 6, 7 and 8 and Fig 3 describe the comparison of the estimated regression coefficient for insulin, fasting glucose levels, and HOMA-IR by serum ferritin groups between the premenopausal and postmenopausal groups. The associations between all three indices and the ferritin groups had a higher level of significance in the postmenopausal group (insulin, p = 0.0005 (model 1), p = 0.0008 (model 2); fasting glucose, p = 0.0277 (model 1), p = 0.0511 (model 2); HOMA-IR, p = 0.0002 (model 1), p = 0.0015 (model 2)). In addition, the postmenopausal group’s estimates increased significantly in the sixth ferritin group compared to those in the first group (insulin, p<0.0001; fasting glucose, p<0.0001; HOMA-IR, p<0.0001). Even after adjusting for all the covariates (i.e., age, alcohol consumption, smoking, exercise, WBC, Hb, Cr, total cholesterol, HRT, and DM treatment), serum ferritin levels were independently associated with insulin, fasting glucose levels, and HOMA-IR in the postmenopausal group (insulin, p = 0.0042; fasting glucose, p = 0.0013; HOMA-IR, p = 0.0046).

**Table 6:** Estimated regression coefficient for insulin by serum ferritin groups.

	Premenopause	Postmenopause	*p*-value
	Estimate(SE); *p*-value	Estimate(SE); *p*-value	
Model 1			0.0005
Ferritin level 1	0	0	
Ferritin level 2	-0.407(0.271); 0.1322	0.144(0.437); 0.7411	
Ferritin level 3	-0.285(0.271); 0.2920	-0.167(0.437); 0.702	
Ferritin level 4	-0.609(0.271); 0.0244	0.216(0.437); 0.6202	
Ferritin level 5	-0.201(0.270); 0.4571	0.615(0.437); 0.1597	
Ferritin level 6	-0.231(0.271); 0.3933	1.754(0.438); <.0001	
Model 2			0.0008
Ferritin level 1	0	0	
Ferritin level 2	-0.778(0.291); 0.0075	0.003(0.439); 0.9941	
Ferritin level 3	-0.604(0.297); 0.0421	-0.249(0.443); 0.5743	
Ferritin level 4	-0.968(0.299); 0.0012	0.068(0.446); 0.8781	
Ferritin level 5	-0.566(0.301); 0.0605	0.196(0.446); 0.6600	
Ferritin level 6	-0.832(0.304); 0.0062	1.287(0.449); 0.0042	

**Table 7:** Estimated regression coefficient for fasting glucose by serum ferritin groups.

	Premenopause	Postmenopause	*p*-value
	Estimate(SE); *p*-value	Estimate(SE); *p*-value	
Model 1			0.0277
Ferritin level 1	0	0	
Ferritin level 2	-1.363(0.873); 0.1183	0.605(1.615); 0.7080	
Ferritin level 3	-1.188(0.873); 0.1733	0.515(1.615); 0.7496	
Ferritin level 4	-1.286(0.873); 0.1407	2.916(1.615); 0.0711	
Ferritin level 5	0.480(0.872); 0.5823	4.521(1.616); 0.0052	
Ferritin level 6	2.722(0.873); 0.0018	7.994(1.620); <.0001	
Model 2			0.0511
Ferritin level 1	0	0	
Ferritin level 2	-2.063(0.809); 0.0108	-0.279(1.325); 0.8333	
Ferritin level 3	-1.903(0.827); 0.0215	-0.375(1.335); 0.7790	
Ferritin level 4	-2.309(0.832); 0.0055	1.712(1.345); 0.2033	
Ferritin level 5	-1.155(0.838); 0.1685	2.179(1.344); 0.1052	
Ferritin level 6	0.428(0.845); 0.6124	4.360(1.355); 0.0013	

**Table 8:** Estimated regression coefficient for HOMA-IR by serum ferritin groups.

	Premenopause	Postmenopause	*p*-value
	Estimate(SE); *p*-value	Estimate(SE); *p*-value	
Model 1			0.0002
Ferritin level 1	0	0	
Ferritin level 2	-0.109(0.074); 0.1406	0.001(0.153); 0.9956	
Ferritin level 3	-0.073(0.074); 0.3227	-0.056(0.153); 0.7126	
Ferritin level 4	-0.153(0.074); 0.0375	0.070(0.153); 0.6463	
Ferritin level 5	-0.006(0.074); 0.9371	0.228(0.153); 0.1363	
Ferritin level 6	0.027(0.074); 0.7117	0.655(0.153); <.0001	
Model 2			0.0015
Ferritin level 1	0	0	
Ferritin level 2	-0.225(0.079); 0.0043	-0.059(0.145); 0.6853	
Ferritin level 3	-0.177(0.080); 0.0274	-0.105(0.146); 0.4742	
Ferritin level 4	-0.279(0.081); 0.0006	0.001(0.148); 0.9945	
Ferritin level 5	-0.139(0.081); 0.0879	0.050(0.147); 0.7344	
Ferritin level 6	-0.185(0.082); 0.0241	0.422(0.149); 0.0046	

HOMA-IR = homeostasis model assessment insulin resistance

Models 1 & 2: multiple linear logistic regression

Model 1 was adjusted for age

Model 2 was adjusted for age + covariates (i.e., alcohol consumption, smoking status, exercise levels, WBC, Hb, Cr, total cholesterol, HRT, and DM treatment)

The graphs describe the least squares (LS) means of insulin, fasting glucose, and HOMA-IR according to serum ferritin groups after adjusting with confounders. The LS means according to insulin levels in premenopausal women were 10.573, 9.794, 9.968, 9.605, 10.007, and 9.741 in the order of increasing ferritin levels. Those of postmenopausal women were 9.842, 9.845, 9.593, 9.910, 10.038, and 11.129. The LS means according to fasting glucose levels in premenopausal women were 129.196, 127.132, 127.293, 126.886, 128.041, and 129.624. Those of postmenopausal women were 115.327, 115.048, 114.952, 117.039, 117.506, and 119.687. Lastly, the LS means according to HOMA-IR in premenopausal women were 3.328, 3.104, 3.151, 3.050, 3.189, and 3.143. Those of postmenopausal women were 2.865, 2.806, 2.760, 2.866, 2.915, and 3.287. Compared to the first ferritin group, all three graphs showed increased numbers in the sixth ferritin group of postmenopausal women, indicating increased risk of IR. (PreM; Premenopause, PostM; Postmenopause)

## Discussion

In this cross-sectional study, we analyzed the associations between serum ferritin levels and various IR-related indices and revealed that elevated serum ferritin levels are strongly associated with increased risk of IR, especially in postmenopausal women. These associations were also independent of several confounders.

Several previous studies describe associations between serum ferritin levels and IR, and there were conflicting results regarding the two genders. Park et al. reported an increasing risk of IR in proportion to the serum ferritin levels of Korean men during a 5-year follow-up period that was independent of other confounding factors, such as age, BMI, smoking status, and hypertension [16]. A Taiwanese study indicated that the relationship between serum ferritin and IR is present in non-diabetic women but not in non-diabetic men [6]. On the other hand, Kim et al. reported that increased serum ferritin levels are associated with IR, type 2 DM, impaired fasting glucose, and metabolic syndrome in men but only with impaired fasting glucose in women [7]. Furthermore, a Japanese study described serum ferritin is associated with markers of IR in Japanese men but not in women [17]. These differences may be due to different study designs with different study populations and adjusting confounders. Although these studies do not agree, we can still consider serum ferritin levels to reflect the development of IR and that there are differences between the genders. Further studies will be needed to clarify the gender differences.

This study only included women and compared the results between premenopausal and postmenopausal women. Postmenopausal women had higher levels of serum ferritin as a result of cessation of menstruation. Obesity and IR indices also increased proportionately to ferritin levels. The results for the obesity indices in the premenopausal group were less clear after adjusting covariates. Guglielmi et al. reported that ferritin concentrations were associated with cholesterol and AST levels in premenopausal women[18]. Consequently, adjusting those covariates might have affected our results.

There are several possible explanations for the relationship between serum ferritin levels and IR. First, several studies have reported that increased iron storage in hepatocytes results in abnormal glucose metabolism [19–21]. Iron-mediated tissue damage is proposed to be initiated by iron-induced oxidant stress [22,23]. Mitochondria are the targets of iron-mediated damage, and iron may be preferentially toxic to cells with high mitochondrial activity [24], such as hepatocytes and pancreatic beta cells. The loss of mitochondrial function leads to developing metabolic syndrome [25]. Therefore, iron-induced oxidative stress combined with mitochondrial defects may be a triggering factor for metabolic disorders, such as insulin resistance.

Second, IR is currently thought to be an inflammatory process. It has been associated with inflammatory factors such as tumor necrosis factor-α and interleukin-6 (IL-6) from adipocytes [26–28]. Serum ferritin is pro-inflammatory, similar to IL-6, which is also correlated with IR [28,29]. Therefore, ferritin and IR may be related to each other via the inflammatory process. Third, lipid peroxidation is suggested to be involved in IR development and it can be induced by iron’s catalytic effects [30,31]. Additionally, iron itself can form highly reactive free radicals that may also disrupt glucose metabolism and cause IR [32,33].

There are some limitations to this study. First, due to the cross-sectional study design, the causality between ferritin and IR is difficult to prove. This study only aimed to show the possible relationship between ferritin and IR, not the mechanism of how ferritin may have affected the development of IR. Second, additional iron markers, such as hepcidin and transferrin concentration/saturation measurements, would have helped differentiate the iron status of the study population. Third, the serum ferritin levels were only tested once for each participant. These levels may have been affected by the participants’ nutritional and health status. It would have been more accurate to measure the ferritin levels more than once with certain time intervals to calculate mean values. Although we did exclude participants who had high WBC levels (indicating inflammatory status), AST, ALT, and liver disease, we did not include the specific nutritional status of the study population with regards to iron consumption.

Despite these limitations, there are strong points in this study. First, to our knowledge, this is the first study comparing the association between serum ferritin levels and IR in premenopausal and postmenopausal women using a large Asian study population (6632 participants). The large sample number allowed us to achieve statistically significant test results. Second, because the data was from a national survey of the common civilian population in South Korea (i.e., not just hospital patients), the risk of having selection bias was reduced. Third, this was the first study to focus on premenopausal and postmenopausal women’s ferritin and IR status. It is interesting to note, from the study results, that postmenopausal women had a higher risk of IR than premenopausal women. The possible explanation for this is that the estrogen production declines at menopause, and this decline increases the levels of oxidative stress [34], which may explain the association between ferritin and insulin resistance.

In conclusion, elevated serum ferritin levels were associated with an increased risk of IR in postmenopausal women. Prospective or longitudinal studies will be needed to further clarify the causality between ferritin levels and IR.

## Supporting Information

S1 File Original KNHANES 2007–2010 data. Original data used for statistical analysis is shown with code specifications.
(XLSX)

## Abbreviations

HOMA-IR: Homeostasis Model Assessment Insulin Resistance
KNHANES: Korean National Health and Nutrition Examination Surveys
HRT: Hormone Replacement Therapy
WC: Waist Circumference
WC/Ht: waist-to-height ratio
LS means: Least Square means
DM: Diabetes mellitus
CVD: Cardiovascular disease
BO: Bilateral oophorectomy
Cr: Creatinine
