# Correction: The Relationship between Serum Ferritin Levels and Insulin Resistance in Pre- and Postmenopausal Korean Women: KNHANES 2007-2010

**DOI:** 10.1371/journal.pone.0160300

**Published:** 2016-07-26

**Authors:** Min Kyoung Kim, Seung Joo Chon, Yeon Soo Jung, Bo Ok Kim, Eun Bee Noe, Bo Hyon Yun, SiHyun Cho, Young Sik Choi, Byung Seok Lee, Seok Kyo Seo

The ferritin values in the second column of Table 1 are written incorrectly. The correct values should appear as 1 (n = 727) F≤9.57. Please see the correct Table 1 here.

**Table 1:** Baseline characteristics of premenopausal women according to serum ferritin groups.

		1 (n = 727)	2 (n = 726)	3 (n = 727)	4 (n = 725)	5 (n = 727)	6 (n = 725)	*p*-value†	*p*-value‡
		F≤9.57	9.57<F≤17.32	17.32<F≤25.86	25.86<F≤36.62	36.62<F≤52.93	F>52.93		
**Ferritin (ng/mL)**		6.2±2.05	13.42±2.26	21.37±2.47	31.14±3.18	44.02±4.6	78.02±29.19		
**Age**		**37.19±8.19**	**35.75±8.23**	**35.64±8.4**	**35.72±8.27**	**36.09±8.16**	**35.91±8.72**	0.0033*	0.0011*
**Ht (cm)**		**158.61±5.83**	**159.24±5.78**	**159.63±5.34**	**159.43±5.37**	**159.1±5.35**	**159.17±5.34**	0.0130*	0.0018*
**BMI (kg/m**^**2**^**)**		**22.43±3.38**	**22.28±3.47**	**22.58±3.55**	**22.37±3.8**	**22.49±3.51**	**22.9±3.84**	0.0205*	0.8424
**WC (cm)**		**75.41±8.6**	**74.9±8.73**	**76±9.15**	**75.14±9.01**	**75.65±8.88**	**77.57±10.2**	< .0001*	0.8470
**WC/Ht**		**0.476±0.057**	**0.471±0.056**	**0.477±0.059**	**0.472±0.058**	**0.476±0.058**	**0.488±0.065**	< .0001*	0.4737
**Fasting glucose (mg/dl)**		**91.59±17.1**	**89.74±11.63**	**89.88±13.92**	**89.81±10.43**	**91.7±17.75**	**93.88±25.62**	< .0001*	0.0633
**Insulin (μIU/mL)**		9.94±4.04	9.57±4.14	9.7±5.18	9.37±4.18	9.77±8.11	9.74±3.98	0.3901	0.0611
**HOMA-IR (mg/dl)**		2.26±1.04	2.15±1.11	2.18±1.36	2.1±1.09	2.25±2.26	2.28±1.18	0.0897	0.0581
**WBC (Thous/μl)**		**5.72±1.14**	**5.89±1.25**	**5.81±1.26**	**5.92±1.25**	**5.99±1.25**	**6.11±1.31**	< .0001*	0.0128*
**Hb (g/dl)**		**12.03±0.996**	**12.99±0.796**	**13.1±0.814**	**13.18±0.768**	**13.24±0.802**	**13.32±0.866**	< .0001*	< .0001*
**Cr (mg/dl)**		0.741±0.105	0.743±0.115	0.734±0.109	0.734±0.111	0.728±0.104	0.73±0.109	0.0657	0.0996
**Total cholesterol (mg/dl)**		**181.18±31.35**	**177.27±30.18**	**174.63±28.68**	**176.55±30.76**	**176.46±31.71**	**179.76±34.3**	0.0007*	0.0014*
**AST (IU/L)**		**17.34±4.54**	**17.46±4.89**	**17.68±5.27**	**17.79±5.12**	**18.24±6.15**	**19.75±7.63**	< .0001*	0.0943
**ALT (IU/L)**		**13.94±6.43**	**14.3±7.11**	**15.03±8.84**	**15.18±7.82**	**16.44±9.7**	**19.03±12.84**	< .0001*	0.0030*
**BMI**	**<25**	576(79.23)	586(80.72)	564(77.58)	571(78.76)	576(79.23)	546(75.31)	0.1974	0.0733
	**≥25**	151(20.77)	140(19.28)	163(22.42)	154(21.24)	151(20.77)	179(24.69)		
**WC**	**<85**	**624(85.83)**	**633(87.19)**	**617(84.87)**	**625(86.21)**	**624(85.83)**	**572(78.9)**	0.0001*	0.0008*
	**≥85**	**103(14.17)**	**93(12.81)**	**110(15.13)**	**100(13.79)**	**103(14.17)**	**153(21.1)**		
**WC/Ht**	**<0.51**	**535(73.59)**	**563(77.55)**	**543(74.69)**	**549(75.72)**	**537(73.87)**	**489(67.45)**	0.0005*	0.0029*
	**≥0.51**	**192(26.41)**	**163(22.45)**	**184(25.31)**	**176(24.28)**	**190(26.13)**	**236(32.55)**		

Tables 2–8 are misaligned in the published article. Please see the correctly aligned tables here.

**Table 2:** Baseline characteristics of postmenopausal women according to serum ferritin groups.

		1 (n = 380)	2 (n = 379)	3 (n = 379)	4 (n = 379)	5 (n = 379)	6 (n = 379)	*p*-value†	*p*-value‡
		F≤29.92	29.92<F≤43.9	43.9<F≤56	56<F≤72.73	72.73<F≤98.21	F>98.21		
**Ferritin (ng/mL)**		19.7±7.03	37.61±3.91	49.86±3.5	64.4±4.92	84.25±7.12	144.24±55.04		
**Age**		**62.35±9.51**	**62.32±8.94**	**61.64±9.2**	**62.61±9.44**	**63.6±9.44**	**65.13±9.65**	< .0001*	0.9667
**Ht (cm)**		**152.1±6.46**	**153.13±6.38**	**153.27±5.92**	**153.18±6.3**	**152.49±5.84**	**152.49±6.15**	0.0432*	0.0171*
**BMI (kg/m**^**2**^**)**		**23±3.42**	**23.31±3.43**	**23.2±2.98**	**23.38±3.41**	**23.51±3.4**	**23.93±3.64**	0.0053*	0.1847
**WC (cm)**		**78.92±9.01**	**80.56±8.93**	**80.14±8.5**	**81.37±8.69**	**81.75±8.95**	**83.62±9.21**	< .0001*	0.0007*
**WC/Ht**		**0.52±0.062**	**0.527±0.059**	**0.523±0.057**	**0.532±0.058**	**0.537±0.06**	**0.549±0.065**	< .0001*	0.0167*
**Fasting glucose (mg/dl)**		**95.39±17.13**	**95.99±19.4**	**95.8±17.62**	**98.35±24.92**	**100.1±22.89**	**103.8±29.21**	< .0001*	0.0902
**Insulin (μIU/mL)**		**9.22±9.4**	**9.36±4.59**	**9.05±3.89**	**9.44±4.02**	**9.84±5.57**	**10.99±6.72**	< .0001*	0.8064
**HOMA-IR (mg/dl)**		**2.25±3.25**	**2.25±1.32**	**2.19±1.3**	**2.32±1.27**	**2.49±1.83**	**2.93±2.75**	< .0001*	0.7501
**WBC (Thous/μl)**		**5.69±1.2**	**5.68±1.21**	**5.73±1.23**	**5.74±1.16**	**5.9±1.29**	**6.02±1.39**	0.0004*	0.4570
**Hb (g/dl)**		**12.79±0.979**	**13±0.82**	**13.18±0.838**	**13.13±0.912**	**13.26±0.936**	**13.36±0.917**	< .0001*	< .0001*
**Cr (mg/dl)**		0.75±0.132	0.741±0.121	0.733±0.121	0.738±0.123	0.739±0.127	0.749±0.133	0.3934	0.1405
**Total cholesterol (mg/dl)**		201.76±34.43	202.61±33.57	204.06±34.86	205.73±37.02	205.18±33.44	207.29±36.3	0.2617	0.0967
**AST (IU/L)**		**21.92±6.52**	**21.68±6.12**	**21.76±6.49**	**22.31±6.41**	**22.89±6.76**	**24.81±9.81**	< .0001*	0.4437
**ALT (IU/L)**		**17.44±7.91**	**17.74±7.81**	**18.29±8.57**	**19.85±9.55**	**20.16±10.36**	**22.51±12.17**	< .0001*	0.0004*
**BMI**	**<25**	**280(73.68)**	**261(68.87)**	**279(73.61)**	**253(66.75)**	**258(68.07)**	**238(62.8)**	0.0083*	0.0014*
	**≥25**	**100(26.32)**	**118(31.13)**	**100(26.39)**	**126(33.25)**	**121(31.93)**	**141(37.2)**		
**WC**	**<85**	**285(75)**	**258(68.07)**	**274(72.3)**	**252(66.49)**	**248(65.44)**	**221(58.31)**	< .0001*	< .0001*
	**≥85**	**95(25)**	**121(31.93)**	**105(27.7)**	**127(33.51)**	**131(34.56)**	**158(41.69)**		
**WC/Ht**	**<0.51**	**166(43.68)**	**157(41.42)**	**162(42.74)**	**142(37.47)**	**122(32.19)**	**105(27.7)**	< .0001*	< .0001*
	**≥0.51**	**214(56.32)**	**222(58.58)**	**217(57.26)**	**237(62.53)**	**257(67.81)**	**274(72.3)**		

**Table 3:** Adjusted odds ratios (95% confidence interval) for BMI (≥25) by serum ferritin groups.

	Premenopause	Postmenopause	*p*-value
	OR(95% CI); *p*-value	OR(95% CI); *p*-value	
Model 1			0.2331
Ferritin level 1	1	1	
Ferritin level 2	0.947(0.731–1.226); 0.6773	1.266(0.923–1.735); 0.1429	
Ferritin level 3	1.150(0.894–1.478); 0.2770	1.001(0.725–1.383); 0.9950	
Ferritin level 4	1.070(0.830–1.379); 0.6005	1.396(1.021–1.908); 0.0366	
Ferritin level 5	1.030(0.799–1.329); 0.8182	1.319(0.963–1.807); 0.0844	
Ferritin level 6	1.295(1.011–1.659); 0.0407	1.676(1.229–2.285); 0.0011	
Model 2			0.3929
Ferritin level 1	1	1	
Ferritin level 2	0.920(0.690–1.225); 0.5673	1.177(0.844–1.642); 0.3368	
Ferritin level 3	1.146(0.860–1.528); 0.3513	0.961(0.683–1.351); 0.8174	
Ferritin level 4	1.077(0.806–1.440); 0.6146	1.238(0.885–1.733); 0.2124	
Ferritin level 5	0.996(0.742–1.337); 0.9799	1.214(0.869–1.698); 0.2560	
Ferritin level 6	1.182(0.884–1.580); 0.2592	1.470(1.055–2.050); 0.0229	

**Table 4:** Adjusted odds ratios (95% confidence interval) for WC (≥85 cm) by serum ferritin groups.

	Premenopause	Postmenopause	*p*-value
	OR(95% CI); *p*-value	OR(95% CI); *p*-value	
Model 1			0.1713
Ferritin level 1	1	1	
Ferritin level 2	0.950(0.700–1.287); 0.7385	1.408(1.025–1.934); 0.0345	
Ferritin level 3	1.160(0.864–1.556); 0.3229	1.158(0.838–1.601); 0.3743	
Ferritin level 4	1.036(0.768–1.398); 0.8148	1.509(1.101–2.069); 0.0106	
Ferritin level 5	1.053(0.782–1.418); 0.7338	1.566(1.144–2.146); 0.0052	
Ferritin level 6	1.728(1.309–2.281); 0.0001	2.09(1.532–2.85); < .0001	
Model 2			0.1713
Ferritin level 1	1	1	
Ferritin level 2	0.840(0.599–1.179); 0.3133	1.326(0.950–1.851); 0.0975	
Ferritin level 3	1.102(0.788–1.540); 0.5709	1.079(0.767–1.519); 0.6613	
Ferritin level 4	0.888(0.631–1.252); 0.4989	1.285(0.915–1.804); 0.1481	
Ferritin level 5	0.876(0.620–1.237); 0.4525	1.414(1.011–1.976); 0.0427	
Ferritin level 6	1.221(0.876–1.703); 0.2386	1.776(1.275–2.475); 0.0007	

**Table 5:** Adjusted odds ratios (95% confidence interval) for WC/Ht (≥0.51) by serum ferritin groups.

	Premenopause	Postmenopause	*p*-value
	OR(95% CI); *p*-value	OR(95% CI); *p*-value	
Model 1			0.3212
Ferritin level 1	1	1	
Ferritin level 2	0.883(0.690–1.131); 0.3261	1.099(0.821–1.470); 0.5261	
Ferritin level 3	1.049(0.823–1.337); 0.7001	1.064(0.796–1.423); 0.6744	
Ferritin level 4	0.985(0.772–1.257); 0.9037	1.291(0.962–1.732); 0.0884	
Ferritin level 5	1.069(0.840–1.360); 0.5900	1.584(1.175–2.137); 0.0026	
Ferritin level 6	1.496(1.182–1.892); 0.0008	1.875(1.380–2.548); < .0001	
Model 2			0.3554
Ferritin level 1	1	1	
Ferritin level 2	0.788(0.598–1.039); 0.0917	1.012(0.742–1.380); 0.9400	
Ferritin level 3	0.964(0.730–1.273); 0.7973	0.988(0.724–1.349); 0.9408	
Ferritin level 4	0.860(0.649–1.138); 0.2906	1.186(0.864–1.629); 0.2919	
Ferritin level 5	0.905(0.683–1.199); 0.4879	1.404(1.018–1.935); 0.0383	
Ferritin level 6	1.102(0.833–1.456); 0.4971	1.498(1.077–2.083); 0.0163	

**Table 6:** Estimated regression coefficient for insulin by serum ferritin groups.

	Premenopause	Postmenopause	*p*-value
	Estimate(SE); *p*-value	Estimate(SE); *p*-value	
Model 1			0.0005
Ferritin level 1	0	0	
Ferritin level 2	-0.407(0.271); 0.1322	0.144(0.437); 0.7411	
Ferritin level 3	-0.285(0.271); 0.2920	-0.167(0.437); 0.702	
Ferritin level 4	-0.609(0.271); 0.0244	0.216(0.437); 0.6202	
Ferritin level 5	-0.201(0.270); 0.4571	0.615(0.437); 0.1597	
Ferritin level 6	-0.231(0.271); 0.3933	1.754(0.438); < .0001	
Model 2			0.0008
Ferritin level 1	0	0	
Ferritin level 2	-0.778(0.291); 0.0075	0.003(0.439); 0.9941	
Ferritin level 3	-0.604(0.297); 0.0421	-0.249(0.443); 0.5743	
Ferritin level 4	-0.968(0.299); 0.0012	0.068(0.446); 0.8781	
Ferritin level 5	-0.566(0.301); 0.0605	0.196(0.446); 0.6600	
Ferritin level 6	-0.832(0.304); 0.0062	1.287(0.449); 0.0042	

**Table 7:** Estimated regression coefficient for fasting glucose by serum ferritin groups.

	Premenopause	Postmenopause	*p*-value
	Estimate(SE); *p*-value	Estimate(SE); *p*-value	
Model 1			0.0277
Ferritin level 1	0	0	
Ferritin level 2	-1.363(0.873); 0.1183	0.605(1.615); 0.7080	
Ferritin level 3	-1.188(0.873); 0.1733	0.515(1.615); 0.7496	
Ferritin level 4	-1.286(0.873); 0.1407	2.916(1.615); 0.0711	
Ferritin level 5	0.480(0.872); 0.5823	4.521(1.616); 0.0052	
Ferritin level 6	2.722(0.873); 0.0018	7.994(1.620); < .0001	
Model 2			0.0511
Ferritin level 1	0	0	
Ferritin level 2	-2.063(0.809); 0.0108	-0.279(1.325); 0.8333	
Ferritin level 3	-1.903(0.827); 0.0215	-0.375(1.335); 0.7790	
Ferritin level 4	-2.309(0.832); 0.0055	1.712(1.345); 0.2033	
Ferritin level 5	-1.155(0.838); 0.1685	2.179(1.344); 0.1052	
Ferritin level 6	0.428(0.845); 0.6124	4.360(1.355); 0.0013	

**Table 8:** Estimated regression coefficient for HOMA-IR by serum ferritin groups.

	Premenopause	Postmenopause	*p*-value
	Estimate(SE); *p*-value	Estimate(SE); *p*-value	
Model 1			0.0002
Ferritin level 1	0	0	
Ferritin level 2	-0.109(0.074); 0.1406	0.001(0.153); 0.9956	
Ferritin level 3	-0.073(0.074); 0.3227	-0.056(0.153); 0.7126	
Ferritin level 4	-0.153(0.074); 0.0375	0.070(0.153); 0.6463	
Ferritin level 5	-0.006(0.074); 0.9371	0.228(0.153); 0.1363	
Ferritin level 6	0.027(0.074); 0.7117	0.655(0.153); < .0001	
Model 2			0.0015
Ferritin level 1	0	0	
Ferritin level 2	-0.225(0.079); 0.0043	-0.059(0.145); 0.6853	
Ferritin level 3	-0.177(0.080); 0.0274	-0.105(0.146); 0.4742	
Ferritin level 4	-0.279(0.081); 0.0006	0.001(0.148); 0.9945	
Ferritin level 5	-0.139(0.081); 0.0879	0.050(0.147); 0.7344	
Ferritin level 6	-0.185(0.082); 0.0241	0.422(0.149); 0.0046	
